# The Uganda Newborn Study (UNEST): an effectiveness study on improving newborn health and survival in rural Uganda through a community-based intervention linked to health facilities - study protocol for a cluster randomized controlled trial

**DOI:** 10.1186/1745-6215-13-213

**Published:** 2012-11-15

**Authors:** Peter Waiswa, Stefan S Peterson, Gertrude Namazzi, Elizabeth Kiracho Ekirapa, Sarah Naikoba, Romano Byaruhanga, Juliet Kiguli, Karin Kallander, Abner Tagoola, Margaret Nakakeeto, George Pariyo

**Affiliations:** 1 Makerere University School of Public Health, College of Health Sciences, Mulago Hill, Kampala, Uganda; 2 Department of Public Health Sciences, Division of Global Health (IHCAR), Nobels väg 9, Karolinska Institutet, SE-171 77, Stockholm, Sweden; 3 Makerere University, Iganga-Mayuge Health & Demographic Surveillance Site, Saza Road, Kampala, Uganda; 4 Saving Newborn Lives, Save the Children, Plot 68/70 Kira Road, Kampala, Uganda; 5 Nsambya Hospital, department of Obstetrics and Gynaecology, Nsambya Road, Kampala, Uganda; 6 International Maternal and Child Health, Department of Women’s and Children’s Health, Uppsala University, Drottninggatan 4, Uppsala, Sweden; 7 Ministry of Health, 6 Lourdel Road, Kampala, Uganda; 8 Department of Health Policy Planning and Management, Makerere University School of Public Health, College of Health Sciences, Mulago Hill, Kampala, Uganda

**Keywords:** UNEST, Newborn, Community health workers, Uganda, Africa, Trial

## Abstract

**Background:**

Reducing neonatal-related deaths is one of the major bottlenecks to achieving Millennium Development Goal 4. Studies in Asia and South America have shown that neonatal mortality can be reduced through community-based interventions, but these have not been adapted to scalable intervention packages for sub-Saharan Africa where the culture, health system and policy environment is different. In Uganda, health outcomes are poor for both mothers and newborn babies. Policy opportunities for neonatal health include the new national Health Sector Strategic Plan, which now prioritizes newborn health including use of a community model through Village Health Teams (VHT). The aim of the present study is to adapt, develop and cost an integrated maternal-newborn care package that links community and facility care, and to evaluate its effect on maternal and neonatal practices in order to inform policy and scale-up in Uganda.

**Methods/Design:**

Through formative research around evidence-based practices, and dialogue with policy and technical advisers, we constructed a home-based neonatal care package implemented by the responsible VHT member, effectively a Community Health Worker (CHW). This CHW was trained to identify pregnant women and make five home visits - two before and three just after birth - so that linkages will be made to facility care and targeted messages for home-care and care-seeking delivered. The project is improving care in health units to provide standardized care for the mother and the newborn in both intervention and comparison areas.

The study is taking place in a new Demographic Surveillance Site in two rural districts, Iganga and Mayuge, in Uganda. It is a two-arm cluster randomized controlled design with 31 intervention and 32 control areas (villages). The comparison parishes receive the standard care already being provided by the district, but to the intervention villages are added a system for CHWs to visit the mother five times in her home during pregnancy and the neonatal period. Both areas benefit from a standardized strengthening of facility care for mothers and neonates.

**Discussion:**

UNEST is designed to directly feed into the operationalization of maternal and newborn care in the national VHT strategy, thereby helping to inform scale-up in rural Uganda. The study is registered as a randomized controlled trial, number ISRCTN50321130.

## Background

Maternal and neonatal illness represent some of the most important health conditions in Uganda and in sub-Saharan Africa (SSA). Globally, of the 7.6 million deaths of children under five years each year [1], 3.6 million (41%) are deaths of babies in the neonatal period, and 98% of these deaths occur in developing countries. Although recent reports show some improvements in maternal mortality globally from the current estimate of 342,900 maternal deaths worldwide in 2008, down from 526,300 in 1980 [2], there has been minimal change in sub-Saharan Africa [2].

Most neonatal deaths, or death within the first month of life, take place on the day of delivery; between 30 and 50% of newborn deaths in Africa are on the first day of life, and 75% occur in the first week alone. The same is true for maternal deaths - approximately 50% of maternal deaths take place within one day of childbirth. A number of factors contribute to this high maternal and newborn mortality including the low (37%) skilled attendance at births, poverty, HIV/AIDS, low-quality antenatal and delivery care, and the unavailability of postnatal care on the continent, as most births occur at home due to problems related to inaccessibility to care.

Despite high use of antenatal care (ANC) (94% for the first ANC visit) in Uganda [3], deliveries at health facilities have remained low (42%), and Emergency Obstetric Care (EmOC) met need is only 14%. Postnatal care (PNC) coverage is very low and maternal mortality, perinatal and neonatal mortality have remained high (maternal mortality ratio 435 per 100,000 live births, perinatal mortality 36 per 1,000 and neonatal mortality 29 per 1,000 live births) [3].

To date, most efforts to improve maternal and newborn health in Uganda have focused on influencing the supply side, usually involving training of health workers, provision of supplies, and health education. Few interventions have been directed at influencing the community or the demand side. Previous efforts in safe motherhood and child health generally neglected newborn care, with the consequence that efforts to achieve Millennium Development Goal (MDG) 4 are currently greatly constrained by a relatively high proportion of neonatal deaths. In addition, focus has been on facility-based care with limited linkages between the facilities and the community.

Based on experiences from Asia, there is now accumulating evidence of the potential for community health worker (CHW) programmes to reduce newborn deaths, even in weak health systems [4,5]. However, there is a lack of adequate successful experiences from sustained and nationwide CHW programmes, especially in SSA. Based on estimates from *The Lancet* Neonatal Survival Series, outreach and family-community care at 90% coverage could avert a substantial number of neonatal deaths [6], suggesting a potential application for programmes aimed at saving newborn lives in weak health systems such as those in sub-Saharan Africa (SSA).

In trial settings conducted mainly in Asia, neonatal mortality was reduced by an average of 30% through home visits by trained community health workers to promote preventive care and/or to provide curative newborn care [7-16]. This strategy of improving maternal/newborn care in low-income countries is also recommended in a joint WHO/UNICEF statement on home visits [16].

There are fundamental gaps in knowledge on how to most effectively implement and scale up community-based interventions linked to facility care, especially in rural poor African communities with weak formal health systems. Hence there is an urgent need to adapt and evaluate culturally and regionally appropriate packages of interventions in the African setting. Community interventions to improve home practices and generate community demand for seeking care need to be matched with an adequate supply of accessible good-quality health services for mothers as well as neonates. Mechanisms to strengthen the community-facility linkage need to be defined, and ways to practically promote care seeking in the neonatal period defined. The adapted intervention package needs to be implemented, and evaluated for effects on desired practices and cost, in order to provide policy makers with information on a scalable intervention package.

## Methods

### Study aim and objectives

To adapt, develop and cost an integrated maternal-newborn care package that links community and facility care, and to evaluate its effect on maternal and neonatal practices in order to inform policy and scale-up in Uganda.

### Specific objectives

#### Formative phase

To inform on the design of a health system-linked, community-based package for mother-newborn, with a feasible delivery mechanism for the Ugandan context.

#### Evaluation phase

To implement and evaluate a community-based maternal-newborn package linked to the health facility.

1. To assess effects related to the package of key outcomes like utilization of maternal and newborn care, key household behaviours and care seeking.

2. To cost the package implementation including cost per household visit, cost per CHW trained, and cost per year of supervision.

#### Dissemination phase

To inform on the design and scale-up of community-based mother-newborn care in Uganda.

**Hypothesis: H1:** Implementation of an integrated care package of five home visits by a CHW during pregnancy and the neonatal period to deliver one-on-one health messages and promote linkage to the health facility improves the following maternal-newborn care practices by at least 20% in two years amongst the target population in rural Uganda:

• % of pregnant women attending ANC at least four times (from 40% to 60%);

• % of pregnant women who get a skilled attendance at delivery (from 30% to 50%);

• % of mothers who put nothing on the cord (to get baseline figures during formative research);

•% of newborns who are managed in skin-to-skin contact after delivery (to get baseline figures during formative research);

• % of mothers and babies who receive postnatal care (from 12% to 50%).

### The study context

Uganda is a land-locked country located in East Africa, and lies to the north along the equator. Uganda has a projected population of 32.4 million people. Landmass is about 241,038 km^2^ in size and the country has a population density of about 137/km^2^. A total of 18% of the country is occupied by open water and swamps, and 12% by forest reserves, game parks and mountains. An estimated 86% of the population live in rural areas, and practise mainly peasant farming. The country’s estimated GDP per capita is US$ 1,300 and 35% of the population is below the poverty line (lives on less than a dollar a day). Per capita expenditure on health is only US$ 12, and more than half of this is out-of-pocket expenditure. The country has a very high birth rate of 47.8 per 1,000 population, leading to a very young and dependant population, with 50% being 14 years and below. Life expectancy is 51.6 years for men and 53.8 years for women.

Since the year 2000, the delivery of health services in Uganda has been decentralized to district level. The Ministry of Health (MoH) plays a stewardship role, providing leadership, standards, funding and policies. On the other hand, districts are responsible for implementation and service delivery. Below the district level are Health Sub-Districts (HSDs), which are administrative zones with about 10 to 20 lower level health facilities. The HSD is headquartered at a health centre level IV (HC-IV), and is structurally a mini-hospital with an obstetric theatre, wards, laboratories, one to two doctors, and several nurses and midwives. However, HC-IVs in the country are operating below standards, with most obstetric theatres being non-functional due to a lack of equipment, personnel or other inputs, or just as a result of neglect. Each HSD has three to four HCs level III (HC-III), and this is the lowest level at which laboratory services, deliveries and management of newborn babies is allowable by national policy.

HCs level II (HC-II) are more accessible to the population, but they are small, outpatient-only units that cannot admit, deliver, perform laboratory investigations, or even treat sick newborn babies. At the lowest level is HC I, which lacks any physical infrastructure. A HC I is basically a collection of community volunteers, together termed the Village Health Team (VHT). They are responsible for community mobilization and linking with the formal health facilities. However, in the majority of districts, most VHTs are not functional.

### Study site

**Figure 1:**
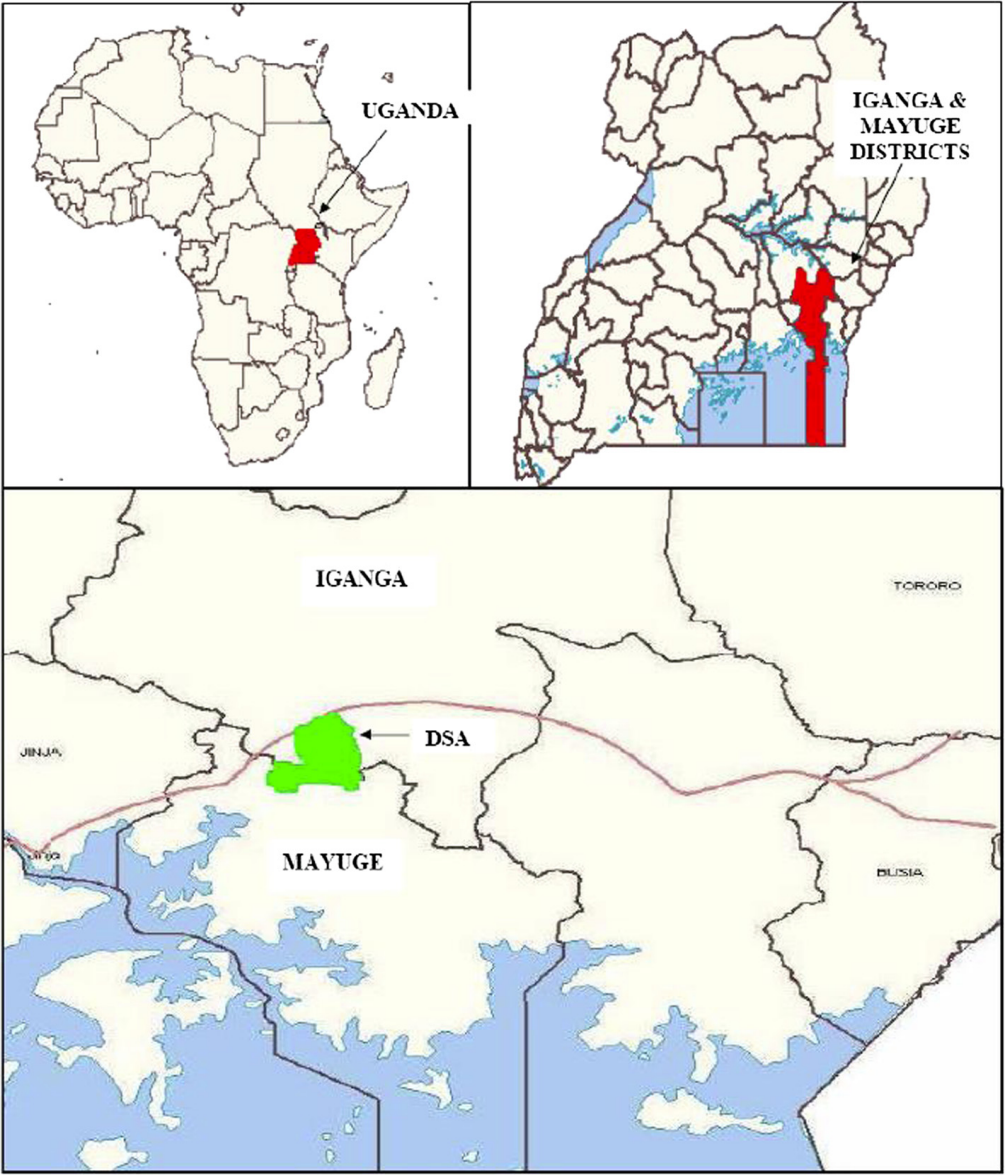
Map showing the location of Iganga-Mayuge districts and the Demographic Surveillance Area, Uganda.

This study will take place in the Makerere University-operated Iganga/Mayuge Demographic Surveillance Site (DSS). It is located in Iganga and Mayuge districts (Figure 1). The DSS is a full member of the International Network for continuous Demographic Evaluation of Populations and Their Health (INDEPTH) A DSS is a powerful ‘population-laboratory’ for efficacy and effectiveness trials.

The Iganga-Mayuge DSS started its operations in 2004 as collaboration between Makerere University and the Karolinska Institutet, Sweden on one hand, and the two host districts on the other, with seed funding from Sida/SAREC. The Demographic Surveillance Area (DSA) has a population of about 67,200 people in 63 villages, 18 parishes and 12,000 households.

Through household visits, field assistants record pregnancies, pregnancy outcomes, deaths and migrations. Since November 2005, village-based demographic scouts notify DSS verbal autopsy interviewers (VAI) of all deaths in the area as they occur on a continuous basis. The VAI visit the bereaved family a month after the event and interview a relative of the deceased who was present at the time of the illness/event and death on verbal and social autopsies (VASA). Three experienced practising doctors independently review the data and assign differential diagnoses on the cause of death according to standard criteria. If there is agreement between at least two of them, the diagnosis is accepted as the definitive cause of death. However, if they do not agree, the three doctors meet to discuss the case and attempt to reach agreement. If this is not possible the cause is coded as undetermined. Besides determination of the most prevalent causes and circumstances of death, we also use the data to profile the burden of different disease, including neonatal conditions. This data from the VASA for maternal and newborn deaths will be analysed as part of the formative research. Results will be used to identify areas in the social processes leading up to death and hence identify modifiable factors in the home or health system on which interventions can be designed.

Iganga Hospital and 13 small health units of which 2 conduct deliveries are located in the DSA. Preliminary findings from the DSA show the following: that 60% of all deaths occur outside a health facility setting; almost all (90%) deaths are due to illnesses as opposed to other causes like injuries and suicide; and the stillbirth rate is about 10/1,000 births, thus indicating under-detection of negative birth outcomes. As it is for most DSS, the first analysis has been a learning experience on the quality of the data, particularly on the reporting of pregnancies, births and newborn deaths.

### Sample and sample size

To evaluate the effect of the intervention, practice differences and changes will be determined as differences in proportions between intervention and control areas. With 80% power and 95% significance level to detect, for example, an increase in attendance four times at ANC from 40% to 60% assuming a coefficient of variation (k) of 0.25 between clusters (parishes), this will require some 60 interviews per cluster in each intervention arm for each practice survey. Similarly, to detect an increase in skilled attendance at birth from 30% to 50%, again with a k of 0.25, some 25 interviews have to be performed. To detect in increase in postnatal care from 12% to 24%, with a k of 0.25, some 30 mothers have to be interviewed. Note that the study is not powered for mortality evaluation. The number of clusters was fixed *a priori* because those were the available ones, and the sample size estimations mainly focused on the cluster size for each outcome for an 80% power.

### Overview of the trial design

#### Randomization

**Figure 2:**
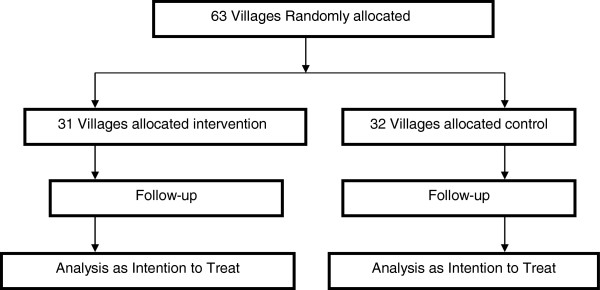
Trial profile.

The unit of intervention and randomization was the village. The study area has 63 villages. All the 63 villages were eligible for randomization. Given the relatively large number of study units (63), we proposed not to stratify or match. Allocating 31 villages to intervention and 32 to comparison we thus have 31 units of intervention, each with 1 to 3 CHWs depending on the number of households therein (Figure 2). Given that the intervention is primarily through home visits by the CHW, we never anticipated contamination to be a big problem, since that would imply making extra home visits. This, however, would be monitored in the annual coverage surveys proposed. Spillover by word of mouth woman-to-woman or other would be captured as a secular trend in the comparison villages. Statistically, we believe it is advantageous to keep the unit of intervention and randomization the same. We also avoid the complexities of which outcome measure to match/stratify on.

### Study procedures

#### The intervention

The intervention was informed by findings from the formative studies [17-20]. For this purpose, an intervention design workshop involving key stakeholders including the Ministry of Health (MoH), Iganga and Mayuge districts, academia, Save the Children, UNICEF and WHO was organized. The package that was agreed on at the end of the workshop was piloted. The intervention has three main strategies:

1. Training and supervising CHWs to dialogue with communities and families during home visits to pregnant and newly delivered women and their newborn babies in the first week after birth;

2. Strengthening linkages between the community and health facilities, including supervision of both health facilities and CHWs in the intervention areas;

3. Training of health workers on essential maternal-newborn care skills and provision of medicines, basic equipment and supplies to health facilities in both intervention and control areas.

### Sensitizations and selection of community health workers

We followed MoH guidelines recommended for selection of members of the VHT. The study team sensitized district staff, traditional birth attendants (TBA), health workers, and private providers about the project. Meetings were organized in each intervention village to sensitize the community on the intended intervention. Following adequate mobilization with active guidance of the project team, the community in a village meeting was then asked to select a CHW who meets the job description for a CHW. In principle, a CHW was selected if he/she met the following criteria: regular/permanent residents, literate, experienced or willing to work as a volunteer, and preferably a mature female already doing some community health work. However, where the community decided to select a man, he was accepted into the project. The community was asked to propose a list of at least three to five potential CHWs from each village and, from this list, the community and project staff were in partnership to select the CHW to work in the village. This was to help avoid any bias that may have existed. Out of those nominated, one CHW per 100 to 150 households was selected to be trained first. The rest of the nominees were kept in the database as reserves in case of drop out. Altogether, 61 CHWs were selected and 58 people were kept in reserve.

The person selected served on the Reproductive Health for the Village Health Team. Other important psychosocial characteristics that have been shown to be important in CHW selection such as empathy; experience of similar problems and situations; respected in the community; considered to be a natural helper or someone that community members would naturally go to in the event of a problem and so on, were used as guiding principles. Any kind of TBA (registered/trained and unregistered/untrained) who was selected by the community was accepted as a CHW and was trained into new community roles other than delivery care, but much more concerned with antenatal care, postnatal care and referral.

### Training of CHWs

All the 61 selected CHWs were trained for five days in essential maternal and newborn care. The training utilized materials adapted for Uganda from the regional workshop on Home-Based Care for Mothers and Newborns organized by UNICEF ESARO in Nairobi, Kenya. The training was conducted by a team of Ugandan trainers, who had attended the training of trainers course, with involvement of staff from the two districts. The training for CHWs was skills-based, and focused on promoting key selected practices as obtained from formative research [17-20] and the recommendations of the workshop.

The 61 CHWs were trained in 3 groups of about 20; the training was non-residential, conducted during May and June 2009 in locations accessible to the CHWs such as health facilities, sub-county headquarters, and churches. Other participants included the CHWs’ supervisors (13 health workers and 4 health assistants) who were in attendance during the training so as to enable linkage of the CHWs to the health workers/health facilities, and facilitate CHW supervision.

Each training session was crowned by the commissioning the participants to start their roles as CHWs in their respective villages of residence. The Local Council 1 (LC1) chairpersons of the villages where CHWs reside were invited for the commissioning so they could introduce the CHWs to their communities. The event was officiated by the local and district leaders of the two districts of Iganga and Mayuge.

### CHW materials and equipment

CHWs were given a set of materials to facilitate their work, give credibility and motivation. They included an identity card; UNEST bag; UNEST T-shirt; notebook; counselling and screening cards; referral forms; registers; birth preparedness forms as well as maternal/child family cards to record appointments and that also have key message reminders on maternal/newborn care. They were also provided with report forms for monthly reports and ‘mama’ or clean delivery kits for demonstration to mothers on the key requirements needed for a clean delivery. ‘Super’ CHWs, who have extra roles as leaders, supervisors and to mobilize fellow CHWs, are provided with a bicycle. All CHWs are paid a token allowance, labelled transport allowance, which was determined during the formative research and conform to Uganda Ministry of Health/UNICEF guidelines for the VHT strategy. An amount of 10,000 Uganda shillings (about US$ 5) is given monthly whenever they attend meetings for supervision, training and so on.

### Home visits by CHWs

Five home visits by CHWs to pregnant women and their babies form the core component of the UNEST study. Two visits are conducted during pregnancy and three during the first week after delivery. The timing and focus of each visit is summarized in Table 1. During the visits, efforts are made to dialogue with the mother, husband and all key people in the family likely to be involved in the pregnancy, delivery and/or postnatal care of the mother and the baby (that is, a family approach).

**Table 1:** Proposed actions during home visits by community health workers

**Two visits during pregnancy (ANC)**	
**Focus of pregnancy visit 1 (as early as possible or in second trimester)**	
●	Counsel on and refer for ANC including TT, IPT and ITNs
●	Counsel on birth preparedness and use of clean delivery practices
●	Assess and counsel on danger signs of pregnancy
●	Counsel on and refer for HIV testing for PMTCT
**Focus of pregnancy visit 2 (in third trimester)**	
●	Counsel on birth preparedness
●	Assess for maternal danger signs and refer if present
●	Counsel on clean delivery practices
●	Counsel on immediate maternal newborn and newborn care practices
●	Counsel on newborn danger signs
**Three visits after pregnancy (PNC)**	
**Postnatal visit 1: birthday to day 2**	
●	Screen for and counsel on maternal and newborn danger signs and refer if present
●	Take newborn’s temperature, weight and respiratory rate
●	Support temperature management (skin-to-skin for all babies, delayed bathing, and wrapping)
●	Support immediate and exclusive breastfeeding
●	Encourage cleanliness especially cord care
**Postnatal visit 2: day 3 after birth**	
●	Assess for maternal and newborn danger signs and refer if necessary
●	Refer for immunization
●	Counsel mother on breastfeeding and birth spacing
●	Reinforce need to seek care/call CHW for signs of local infection or danger signs
**Postnatal visit 3: day 5 to 7 after birth**	
●	Assess for maternal and newborn danger signs and refer if necessary
●	Refer for immunization
●	Counsel mother on breastfeeding and birth spacing
●	Reinforce need to seek care/call CHW for signs of local infection or danger signs
●	Promote access to under five clinics and family planning at six weeks
**If very low birth weight suspected**^**1**^	
●	Refer if also danger sign present or two extra visits to support home care (breastfeeding, warmth, early danger sign recognition) if no danger sign or referral not possible
●	Promote temperature management (skin-to-skin, wrapping and delayed bathing)
●	Assist with feeding if needed
●	Attention to hygiene
**Additional home visits: when called by caretakers**	
●	Check for signs of local infection and danger signs
●	Give early treatment (tentatively cotrimoxazole) and arrange facilitated referral

^1^In Ghana, maternal sensitivity was 73% and specificity 93% to detect birth weight <2 kg. Source: Final Report of the NEWHINTS Formative Research. ANC, antenatal care; IPT, intermittent presumptive treatment; ITN, insecticide-treated net; PMTCT, preventing mother-to-child transmission; PNC, postnatal care; TT, tetanus toxoid immunization.

The CHWs use a counselling and problem-solving approach concerning key gaps in care practices identified during the formative research. The CHW advises mothers on ideal family care practices like immediate breastfeeding, keeping baby warm by skin-to-skin care and proper wrapping, delayed bathing as well as appropriate cord care. The CHW further assesses and screens for danger signs all babies and mothers at each of the three postnatal visits and facilitates referral to health facility any baby or mother found with danger signs. They write a referral note, which has a feedback section that is filled in by the health worker and sent to the CHW. CHWs conduct follow-up visits for referred cases within 24 hours, and an additional at least two postnatal visits to low birth weight (LBW) babies in the second week of life of the baby.

### Supervision of community health workers

The overall supervision is conducted by the district health teams (DHTs) of Iganga and Mayuge Districts led by the district health visitors (DHVs) in accordance with the existing district health service structure with support from the Uganda Newborn Survival Study (UNEST). Each CHW is assigned to a nearby health unit as the primary unit of referral but also the unit that will supervise her monthly.

The district supervisors, with support from UNEST, supervise the health centers/health workers using a checklist that has been developed in accordance to the national newborn standards of care

The DHVs facilitate CHWs’ group meetings with the aim of encouraging CHWs to discuss common problems and successes so that they can learn from and support each other (peer-to-peer learning). The group supervisions are held at the training sites close to the community, and involve the study team members and the CHWs’ supervisors. These meetings initially have been on a monthly basis to help the CHWs become confident and more knowledgeable.

Health workers hold individual discussion sessions with CHWs on a monthly basis at the health facility to provide support, motivation and to solve problems. Once a month, the supervisors accompany CHWs on their visits to observe how they are doing (directly observed supervision (DOS)) and provide feedback, which helps solve any problems and provides support and motivation. The health worker liaises with community leaders to solicit their opinions about and support for the work of the CHWs. This helps ensure the CHWs feel accountable to the community and that the community knows the CHW is working for them.

‘Super’ CHWs (leaders of CHWs per parish/given number of CHWs) coordinate the work of fellow CHWs in their respective parishes and communicate to the health workers any developments in the community as well as giving feedback to the CHWs.

### Health facility strengthening and district health team collaboration

The formative research identified a lot of inadequacies in the current provision of maternal/newborn care in the health facilities [17,18]. Efforts have been made to ensure that all facilities in and around the study area were strengthened through training of the health workers, provision of a one-off catalytic supplies and medicines as well as collaboration with the DHT to continuously provide the essential basic requirements for care of mothers and neonates. All facilities that conduct deliveries were supplied with partographs to monitor the progress of labour. In addition, routine support supervision using national maternal/newborn standards of care is done by the DHT and project staff.

In the hospital, the records system was improved through provision of admission files to the maternity ward so as to track the care being given to mothers and their babies. A kangaroo mother care (KMC) and special care rooms were also initiated in the hospital by modifying existing structures to improve care of the high-risk newborn babies. Two midwives were supported to rotate in the neonatal care unit in Mulago National Referral Hospital for two months. A consultant obstetrician with expertise in maternal and perinatal audit sensitized staff of Iganga hospital on how to operationalize the audit cycle [21].

**Figure 3:**
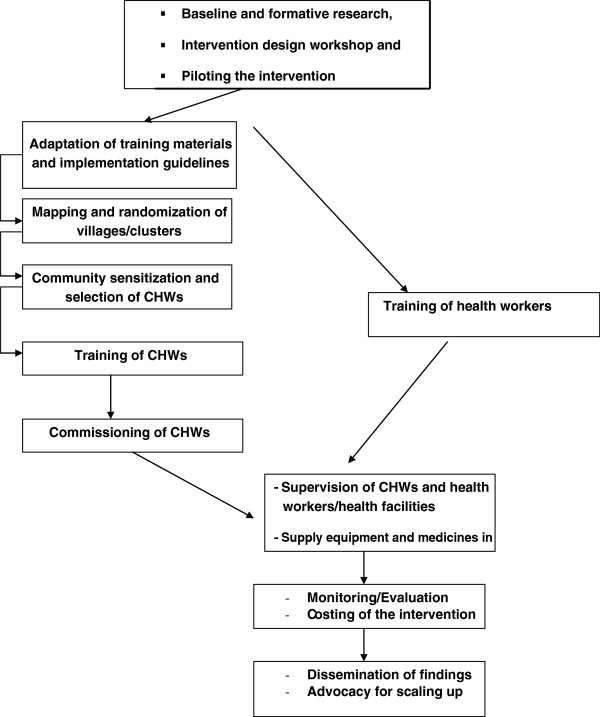
The process taken to operationalize the intervention.

Figure 3 summarizes the process taken to operationalize the intervention.

### Pathways to effect in intervention

If the educational components are successful in changing behaviours associated with risk in neonates, then the interventions should result in improvement in neonatal mortality (Table 2). Hopefully, the combination of improved health facility services and increased awareness and support at the community level, will result in greater compliance with pre- and postnatal care recommendations in a situation where the quality of the services holds minimum standards, thus involving components of both the ‘demand’ and ‘supply’ side. This is the model and assumption being tested in this study.

**Table 2:** Proposed actions, targeted cause of death and behaviour

**Intervention**	**Cause of death addressed**	**Behaviour addressed**
**Community worker: pregnancy visit 1 and 2**		
**Community health worker: pregnancy visit**		
• Counsel on and refer for ANC including TT, IPT and ITNs	• Neonatal tetanus, risk associated w/ maternal malaria	• Low four-visit ANC rate, low malaria treatment rate
• Counsel on birth preparedness and use of clean delivery practices	• All causes	• Poor delivery practices
• Assess and counsel on danger signs of pregnancy	• Prematurity, infection	• Low HF use rate for emergencies
• Counsel on and refer for HIV testing for PMTCT	• HIV/AIDs transmission	• Lack of understanding of HIV/AIDs
• Introduce key neonatal behaviours such as immediate initiation of breastfeeding, delayed bathing, immediate wrapping, skin and cord hygiene, and skin-to-skin care	• All causes	• Poor delivery practices
**Pregnancy visit 2**		
• Counsel on birth preparedness	• Infection	• Low HF use, lack of awareness
• Assess for maternal danger signs and refer if present	• All causes	• As above
• Counsel on clean delivery practices	• Infection	• Lack of awareness
• Counsel on immediate maternal and newborn care practices		• Lack of awareness
• Counsel on newborn danger signs		
**Community worker: postnatal visit 1 and 2**		
**Postnatal visit 1: birthday to day 2**		
• Screen for and counsel on maternal and newborn danger signs and refer if present	• Prematurity, infection	• Lack of HF use, lack of awareness
• Support temperature management (skin-to-skin for all babies, delayed bathing, and wrapping)	• Risk from low birth weight, infection	• Lack of HF use
• Support immediate and exclusive breastfeeding	• Hypothermia	• Low HF use for deliveries
• Encourage cleanliness especially cord care	• Overall risk, infection	• Lack of compliance
	• Infection	• Low HF use for deliveries
	• Prematurity, infection	• Low awareness
	• Hypothermia, infection	• Low HF use for deliveries, awareness
	• Risk from immunizable diseases	• Low TT and other EPI rates
	• Overall risk, infection	• As above
**Postnatal visit 2 and 3: day 3 and day 5 and 7 after birth**		
• Assess for maternal and newborn danger signs and refer if necessary		
• Refer for immunization		
• Counsel mother on breastfeeding and birth spacing		
**If very low birth weight suspected**^**1**^		
• Refer if also danger sign present or two extra visits to support home care (breast-feeding, warmth, early danger sign recognition) if no danger sign or referral not possible		
• Promote temperature management (skin-to-skin, wrapping and delayed bathing)		
• Assist with feeding if needed		
• Attention to hygiene		
**Health facility strengthening**		
• Training	• Sepsis	• Poor quality of care, low HF use for deliveries, inadequate equipment
• Provision of supplies and medicines	• Sepsis	
• Improved sepsis management at lower HF	• Sepsis	
• Supervision and monitoring	• Overall risk	

EPI, expanded programme of immunization; IPT, intermittent presumptive treatment; ITN, insecticide-treated net; HF, health facility; PMTCT, preventing mother-to-child transmission; TT, tetanus toxoid immunization.

The way that these interventions are likely to affect neonatal mortality from infection is by reducing the risk of infection, improving the management of infection, improving the timing of management of infection, improving compliance with treatment. These improvements would likely reduce the proportion of neonatal deaths from infection significantly. We will monitor the different components to be sure that there is solid understanding of how mothers respond to the community visits, and how facilities respond to referred infants. With regard to other causes of neonatal death, birth asphyxia may be improved by increasing attendance at deliveries by skilled attendants, and by identification of risk pregnancies - with appropriate referral. Neonatal deaths from prematurity and low birth weight can be prevented by early identification of risk infants, and appropriate care at home and at a facility.

### Process documentation and monitoring

We attempted to separate the roles of the ‘implementers’ and the ‘evaluators’ in this study. The ‘implementers’ are CHWs and health workers documenting the supervision process and abstracting information from the CHW registers and health facility records. This formed the proposed monitoring part of the intervention, to be part of the product that is evaluated and may be potentially scaled-up.

On the ‘evaluator’ side, process documentation used both a descriptive ‘process documentation’ and quarterly ‘adequacy survey’ in order to enable us to interpret why the intervention was effective or not and will provide very useful policy-relevant information regarding replicability, transferability and how to improve the intervention.

At the initiation of the study, one full-time staff was recruited as documentation officer. She recorded all key events that may affect the outcome of the intervention like drug stock-outs/availability, staff transfer, training, and protocol amendments on a regular basis in order to assist replication in other sites and scale-up. In addition, we also collect data on contextual factors about the setting into which the intervention is being implemented. This is because different settings will influence the extent to which the intervention works or does not work. By collecting information on the different settings in the clusters then any differences in impact can be interpreted in relation to these characteristics. This supplements the traditional process documentations that will be conducted. The understandings developed through these ‘process evaluations’ help to improve interventions and, most importantly, will help transfer and replicate them effectively in other settings. To this end, we will, for example, be using supervisory records more in the evaluation (especially the process evaluation).

The following is used as sources of data for the monitoring and evaluating (M & E) process:

1. CHW register - this documents pregnancies; home visits during pregnancy and after birth records; referrals made and time of completion (routine data monitoring)

2. Health facility registers and health management information systems (HMIS) reports (routine data monitoring)

3. Household surveys and health facilities surveys The data from process evaluation is designed to directly inform improvements in implementation.

Again, whereas a number of process indicators are monitored (Table 3), the key specific ones include the following:

**Table 3:** Process indicators

**Area**	**Process indicators**
**Community Health Worker (CHW) competence and effectiveness**	• % of pregnant women seen by a CHW once
	• % of pregnant women seen by a CHW twice
	• % of mothers seen in first 24 hrs after birth
	• % of newborns seen in first 24 hrs after birth
	• % of all home births seen by a CHW twice in the first week
	• quality of CHW records in terms of completeness and timeliness
	• client satisfaction with CHW activities
	• knowledge and skills of CHW on maternal and newborn care
	• CHW satisfaction with home visiting activities
**Health facility**	• health facility utilization for ANC, deliveries and newborn care
	• % of babies managed according to IMNCI guidelines
	• health workers’ knowledge and skills in managing pregnant and newly delivered women and sick newborns according to adapted maternal and newborn guidelines
	• essential drugs and supplies availability/stock-outs
	• health workers’ satisfaction
	• number of planned joint meetings with CHWs held
	• number of planned supervisory visits of CHWs conducted
**Linkages between community and health facility**	• % of women with childbirth complications identified at home who reach a facility
	• % of babies seen at home with danger signs who are referred reaching the facility
**Sustainability**	• CHW retention and turnover

ANC, antenatal care; IMNCI, Integrated Management of Neonatal and Childhood Illness.

CHW competence and effectiveness:

– % of pregnant women seen by a CHW twice

– % of mothers seen in first 24 hrs after birth

– % of newborns seen in first 24 hrs after birth

– % of all home births seen by a CHW twice in the first week

– linkages between community and health facility

– % of babies seen at home with danger signs who are referred and reach the facility.

### Study outcomes

The primary evaluation of this study will include intermediate outcomes and process indicators. Neonatal mortality will be followed during the study in intervention and control areas as a secondary outcome, but the study is not powered to show mortality impact within two years. Whereas a number of intermediate outcomes will be evaluated in this study, the key ones will be as follows:

### Primary outcomes

1. In ANC

– % of pregnant women attending ANC two, four or more times

– % of pregnant women who know at least two danger signs of pregnancy

– % of pregnant women who prepare for birth

2. In the intrapartum period

– % of pregnant women who have a skilled attendant at delivery

– % of women who went to the HC in an emergency

3. In the postnatal period

– % of babies who are initiated on breast-feeding in the first six hours of birth

– % of babies who are exclusively breastfed during the neonatal period

– % of babies whose first bath was delayed for six and twenty-four hours

– % of mothers who put nothing on the cord

– % of mothers who know at least three neonatal danger signs

– % women whose children were managed in skin-to-skin contact after delivery.

– effectiveness of sepsis management (special studies to determine: maternal and CHW knowledge, compliance and timing of referral, and adequacy of treatment following referral)

Table 4 outlines in details all intermediate outcomes that will be evaluated in the study.

**Table 4:** Study outcomes

**Area in the continuum of care**	**Outcome to be evaluated**
	**NB. Analysis to be done separately for home and health facility births**
Antenatal care	• % of pregnant women attending ANC two, four or more times
	• % of pregnant women who know at least two danger signs of pregnancy
	• % of pregnant women who prepare for birth*
	• IPT in pregnancy
	• ITN use in pregnancy
	• Tetanus toxoid coverage
	• Uptake of VCT % of mothers who tested for HIV during pregnancy
	• Uptake of IPT - by number of doses
Intrapartum care	• % of pregnant women who have a supervised delivery
	• % of pregnant women who deliver at a health unit
	• % of babies whose cord was cut with a clean instrument
	• % of babies who are immediately dried at birth
	• % of babies who are immediately wrapped after birth
	• % of babies who are born on a clean surface
	• % of home births attended by two assistants
	• % of women who went to the HC in an emergency
Postnatal care	• % of babies whose cord was cut with a clean instrument
	• % of babies who are initiated on breastfeeding within one and twenty-four hours of birth
	• % of babies who are exclusively breastfed during the neonatal period
	• % of babies whose first bath was delayed for six and twenty-four hours
	• % of mothers who put nothing on the cord
	• % of mothers who know at least three neonatal danger signs
	• % of babies who are immediately dried at birth
	• % of babies who are immediately wrapped after birth
	• % of babies who are born on a clean surface
	• % of home births attended by two assistants
	• % women whose children were managed in skin-to-skin contact after delivery
	• mothers who received counselling regarding family planning by six weeks postnatally
	• % of babies who were taken for care if they were ill
	• % of babies referred to health facility by CHW that reach, and timeliness of reaching
Impact level	newborn deaths and stillbirths (note not powered to measure significant reduction in NMR) but we will explore each maternal and newborn death using the VASA and case–control study for, for example, intervention efficacy.
	Neonatal mortality rates will be calculated by intervention and comparison areas

*Birth preparedness will be operationalized through having made plans for where to deliver, transport, and preparing baby/delivery materials. IPT, intermittent presumptive treatment; ITNs, insecticide-treated nets; NMR, neonatal mortality rate; VASA, verbal and social autopsies; VCT, voluntary counselling and testing.

### Data collection for quantitative surveys

The DSS data collection and recording system will be used. This is a comprehensive system in which all households have been mapped and given a unique number. All members of each household are known. Each village has a trained data collector and every five to six field assistants have a field supervisor and a local community key informant. A team of local enumerators have already mapped the 18 parishes using geographical information systems (GIS), identified and allocated a unique identification number to every household (defined as a group of individuals sharing one kitchen), did a baseline census of demographic and socio-economic indicators, and generated a list of female household members according to predefined written protocols. During the thrice-yearly update rounds questions are asked of all females 12 to 50 years of age, whether they are pregnant and approximate age of the pregnancy estimated. This pregnancy registration is then followed up in the next update round for birth outcome. In addition, community key informants report births to the DSS staff.

Repeated surveys of practices are done twice per year. Each survey takes some three months to complete. Based on pregnancy recording from the DSS round and birth reports by community key informants at the estimated end of the neonatal period, a field worker will visit the woman to interview on practices during pregnancy, delivery and the first month of life. The interview will include details of any preceding pregnancy, home-care practices, exposure to the CHW home visits, and use of health services for neonatal illness. The questionnaires will be written in English and translated into the local language, Lusoga, and the accuracy of the translation will be checked by back translation into English. Medical experts will review the questionnaire for content validity. Interviewers will administer the survey in Lusoga, the local language. The survey will be pilot tested among 20 mothers outside the DSA and the resulting suggestions regarding clarity and cultural appropriateness will be incorporated.

Field editing will be done. After correction, data will be double-entered into a relational database management system.

### Data analysis

The detailed analysis plan for the trial will be finalized during the evaluation preparation phase. For the qualitative study, content analysis will be used for data analysis and emerging themes arranged into categories. NVivo version software (QSR International, Melbourne Australia) will be used for the analysis.

Quantitative analysis of practice variables will use descriptive univariate analysis for all variables separately, then an intention-to-treat approach comparing summary variables in the intervention to control clusters. However, the effects of the health facility strengthening will be assessed in the control clusters using a ‘before-after’ approach. Outcomes will be compared with adjustment for clustering using appropriate Stata commands (svy). For 2×2 cross tabulations containing cells with expected frequencies of fewer than 5, statistical significance will be determined using Fisher’s exact test, the method consists of evaluating the sum of probabilities associated with the observed table and all possible 2×2 tables that have the same row and column totals as the observed data but exhibit more extreme departure from independence; Yates’ corrected chi square will be used for all others when testing for independence in a contingency table. For cross tabulations with greater than 2 rows, statistical significance will be determined using the Pearson chi square test.

Analysis of variance (ANOVA) will be used for statistical comparison of means. Randomized experiments suggest that ANOVA is particularly appropriate for the analysis of data from experiments in which each subject/individual in the experimental unit is randomly assigned to one of two or more different treatment conditions (control and intervention groups). All subjects in a particular group will receive the same treatment, and differences in the effects of the treatments (control and intervention) are expected to produce differences between groups in post-treatment scores on a relevant measure.

In midterm evaluation, in order to see who is and who is not receiving the intervention, we will carry out analyses using important criteria such as: reach/coverage (who is receiving and not receiving the intervention and what are their characteristics) (data to come from the surveys we are planning from ‘exposure to CHW intervention’ variable); and fidelity (the extent to which the intervention is delivered as originally defined.

### Hypothesis testing

The primary analysis for each outcome will be intention-to-treat, which will be defined by a woman’s village of residence. All analyses will account for the cluster-randomized design using random effects logistic regression and will be carried out both with and without adjustment for potential confounders. The estimated effect of the intervention will be presented as a relative risk together with a 95% confidence interval (CI). Logistic regression will be used to explore whether the hypotheses stated below are true or not, and each will be adjusted for intraclass correlation (ICC), coefficient of variation (k), socio-economic status and other potential confounders. The hypotheses are that: the intervention increases

1. % of pregnant women attending ANC at least four times (from 40% to 60%)

2. % of pregnant women who get a skilled attendance at delivery (from 30% to 50%);

3. % of mothers and babies who receive postnatal care (from 12% to 50%).

### Additional analyses

GIS and socio-economic data are already being collected in the DSS and thus provide a good opportunity to undertake these analyses. In the control area, data analysis will also be done by doing a before-and-after comparison to estimate the effect of only health facility improvement on maternal and neonatal care facilities. Subgroup analyses estimating separate intervention effects for facility- and home-based deliveries.

Analysis will also be done taking account of the quality of the intervention received, as assessed by the process indicators and number of visits received. Because some study participants are highly mobile and some women will move from their place of residence to their parents’ or in-laws’ houses for delivery of the baby, it is envisaged that there may be significant movements across the arms of the trial (that is, some women will move from intervention to control areas and vice versa) during the intervention period. This movement can be tracked using our surveillance system. Thus we will also compare outcomes subdividing women and newborns into the following groups: whether the visits were only pre-delivery or only post-delivery or both, and whether the woman received all five visits, only four, three or two or less visits. However, the main analysis will be based on intention to treat so women who are enrolled in an intervention area, but move to a control area for delivery, will be included in the intervention group.

In addition, analysis will also be done of the mean practice score (a numerical outcome) will be carried out using random effects (multilevel) models that explicitly model the similarity between individuals in the same cluster; (these are not suitable for binary outcomes).

For all statistical determinations, significance levels will be established at 5% level of significance. Coefficient of variation will be reported.

While not a primary outcome, variable stillbirth rate and neonatal mortality rate will be followed through the study in both intervention and control areas. We will define miscarriage as cessation of a presumptive pregnancy before 28 weeks of gestation and stillbirth as fetal death after 28 weeks of gestation but before delivery of the baby’s head, which was a modification of the 22-week definition to meet local practicalities. We will classify neonatal death as death of a live born infant within 28 completed days of birth. Early neonatal deaths will refer to deaths within 7 completed days after delivery. Separate analysis will be done for neonatal mortality excluding the first day of life in order to assess the role of sepsis.

### Equity

This study is by nature a pro-poor/marginalized group intervention. In addition, to ensure equity in access to the intervention, we will determine, through small rapid surveys to determine who is and who is not receiving the intervention, and adjust accordingly. While this intervention intends to reduce barriers to seeking care similar to other ‘pro-poor’ interventions inequities in intervention uptake and service utilization are foreseen. We will analyze uptake of the intervention and home-care practices by socio-economic group using already collected DSS data on assets and education. Using the ‘standard’ approach of asset quintiles, we will look for differences in uptake and outcome. Depending on the socio-economic differences in results of the baseline practice survey, we may undertake a study of equity-specific barriers to care.

### Economic evaluation

The costing approach will be undertaking both programmatic and cost-effectiveness analysis. The economic evaluation will be divided into the costing and cost-effective analysis (CEA) to be undertaken from a provider’s perspective. The costs will be measured prospectively using the COIN tool developed by Save the Children. The outputs of the intervention will be calculated (that is, cost per visit and cost per woman). In order to inform scale-up process, the costs will be presented as set-up costs (one-off and repeatable) and implementation costs. Repeatable set-up costs will include recruitment and training of CHWs and their supervisors. In evaluating costs of human resources, the time use of CHWs will be captured in detail to determine their effectiveness and workload. Health facility costs will also be captured including recurrent cost items consumed, and staff time will be measured.

### Ethical considerations

The trial protocol was approved by Makerere University and the Uganda National Council of Science and Technology. In addition, approval was sought from the district authorities and local leaders in the communities where the study is being conducted. For the intervention and mortality surveillance, which are applied at community level, consent is sought from the individual woman, who of course is free to accept or refuse, for example, intervention home visits.

This intervention is not invasive. Therefore, no marked risks exposed to patients. For the community, the most immediate benefit is improved knowledge and care of newborn babies and their mothers. Efforts have been made to improve health units to support referral in both intervention and control areas.

For the evaluation component informed consent will be requested from study subjects, the local community and confidentiality will be assured. Patient charts and any personal information of study subjects will be restricted to the medical care staff and the investigators directly involved in the study. During the study period, anybody in the community found sick by the study team is referred appropriately.

At the end of the study, if the community intervention is proven effective, we will extend the project to the control areas and possibly to the rest of the two districts with support from the districts themselves. A mechanism for this will be discussed with the districts.

The study has a Data Monitoring and Advisory Board whose members were identified from local experts. The DSMB meets annually. The study has been registered as a randomized controlled trial both locally and internationally (ISRCTN50321130; Ref CCT-NAPN-19173).

### Dissemination of trial findings

Trial findings will be shared promptly with the Technical Steering Committee, and discussed with the local District Health Teams. Local dissemination meetings with the study populations will be held. A CD will be compiled containing all intervention materials plus a detailed implementation evaluation report of lessons learned and shared widely. Policy briefs will be prepared and circulated nationally and internationally to relevant policy and donor organizations, and if possible a national workshop held to discuss the findings, lessons learnt concerning implementation and policy implications.

Trial findings will also be disseminated in scientific meetings and papers on: the impact of the intervention on neonatal mortality; impact on neonatal care practices; any intervention differences by place of delivery or between rural and urban villages; process outcomes, and lessons learned concerning working with volunteers, supervision, monitoring performance; training volunteers to assess babies and how well do they do; strategies to promote coverage; factors influencing response to specific care recommendations including special care for low birth weight babies and referrals; and cost-effectiveness of the intervention.

Requests to analyze or publish data from persons external to the study will be entertained three years after the databases are frozen. The requesting researcher, in addition to at least two persons from within the project team, will author such publications and acknowledgement will be given to the project team including the collaborators.

## Discussions

This study is an effectiveness study designed to directly influence policy. Therefore implementation will be done in close consultation with policy makers and there will be ongoing feedback with preliminary findings. In addition, we will also share the study materials with all interested parties even well before the final evaluation is made. There will be close documentation of all implementation steps, challenges, innovations and experiences occurring at both the demand and supply sides. In addition, the study will be costed as this is important for influencing the policy agenda.

The study has some limitations mainly related to lack of power to measure mortality impact. Another limitation is that the study is implemented in a small area where the risk of contamination is high.

## Trial status

The study is already underway and final evaluation and results should be available late 2012/early 2013.

## Abbreviations

ANC: antenatal care; ANOVA: analysis of variance; CEA: cost-effective analysis; CHW: Community Health Worker; CI: confidence interval; DHT/V: District Health Team/Visitor; DSA/S: Demographic Surveillance Area/Site; GIS: geographical information system; HSD: Health Sub-District; INDEPTH: International Network for continuous Demographic Evaluation of Populations and Their Health; IPT: intermittent presumptive treatment; LBW: low birth weight;M&E: monitoring and evaluation; MDG: Millennium Development Goals; MoH: Ministry of Health; NMR: neonatal mortality rate; SSA: sub-Saharan Africa; TBA: traditional birth attendantVAI, verbal autopsy interviewers; VASA: verbal and social autopsies; VHT: Village Health Team

## Competing interests

The authors declare that they have no competing interests.

## Authors’ contributions

The paper was drafted by PW and GN; all authors reviewed the paper, approved the final manuscript, and had major inputs to the trial design and intervention development.
